# Cigarette smoking and breast cancer risk: a population-based study in Sweden

**DOI:** 10.1038/sj.bjc.6604007

**Published:** 2007-10-02

**Authors:** C Magnusson, S Wedrén, L U Rosenberg

**Affiliations:** 1 Division of Social Medicine, Department of Public Health Sciences, Karolinska Institutet, Stockholm 171 76, Sweden; 2 Department of Medical Epidemiology and Biostatistics, Karolinska Institutet, Stockholm 171 77, Sweden

**Keywords:** smoking, alcohol consumption, breast cancer, aetiology, epidemiology

## Abstract

In a Swedish population-based case–control study, smoking showed no convincing association with risk of postmenopausal breast cancer – regardless of timing or level of smoking exposure – either overall or among subgroups.

The role of smoking in breast cancer aetiology has been extensively studied (Band *et al*, 2002; Collaborative Group on Hormonal Factors in Breast Cancer, 2002; Terry and Rohan, 2002; Lawlor *et al*, 2004). Yet, the association remains equivocal and much debated (Terry and Rohan, 2002; Johnson, 2005). Smoking has been proposed to increase breast cancer risk, based on studies showing breast epithelial genotoxicity of tobacco-related compounds (Morabia, 2002), but also to exert an ‘anti-oestrogenic effect’ and thence to reduce risk (Terry and Rohan, 2002).

We present results from a large population-based study, on the associations between the hypothesised ‘carcinogenetic mode’ of smoking, that is smoking prior to first birth or among nulliparous women, *vs* the ‘anti-oestrogenic mode’, that is recent smoking and risk of breast cancer.

## MATERIALS AND METHODS

### Study population

We conducted a case–control study of breast cancer among all Swedish-born women aged 50–74 years, in Sweden between October 1993 and March 1995.

Cases were women with an incident invasive breast cancer, identified through the six Regional Cancer Registries. Out of 3979 eligible cases, 3345 (84%) participated. Controls were age-frequency matched according to the age distribution of cases, and randomly selected from the study population, using the Registry of the Total Population. Of the 4188 eligible controls, 3454 (82%) participated in the study.

### Data collection

Study participants completed a postal questionnaire on average 4 months after diagnosis. Participants were asked in detail about their life-course smoking history and about other established breast cancer risk factors. Telephone interviews were conducted among controls who declined completion of the postal questionnaires. Out of all participating controls, 14% contributed information in this manner.

### Classification of smoking history

Women were defined as ever smokers if they had smoked a total of at least 100 cigarettes, or if they had smoked regularly for at least 1 year. Women were attributed a 10-year duration of smoking for each 10-year age period they reported to be smokers, if they smoked during age-periods before and after. If not, they were attributed a duration of 5 years for each 10-year age period they reported to be smokers. Lifetime pack-years was calculated as the average smoking intensity multiplied by the estimated smoking duration.

### Statistical analyses

Relative risks were estimated using unconditional logistic regression, adjusted for age in 5-year categories, yielding odds ratios (ORs) and 95% confidence intervals (CIs). Possible confounders were included as covariates in age-adjusted models one at a time and included in the final models if they affected parameter estimates more than 10%. The tested covariates were age at first birth, body mass index, socioeconomic position, alcohol intake 1 year before data collection, age at menarche and menopause, parity, use of menopausal hormone therapy, age at menopause, family history of breast cancer and benign breast disease. The final models were adjusted for age, age at first birth, body mass index and alcohol intake.

Linear trends and effect modification were assessed using Wald tests.

Analyses were performed using SAS system, version 9.1 (SAS Institute, Carey, NC, USA). All statistical significance levels (*P*-values) quoted are two-sided.

## RESULTS

The prevalence of smoking was similar among cases and controls, with 24 and 23% being current smokers, and 22 and 20% being past smokers, respectively. The distribution of established breast cancer risk factors varied significantly with smoking status, such that ever smokers were younger, taller, thinner, consumed more alcohol, had on average an earlier age at menarche, first birth, and menopause, and had used menopausal hormone therapy to a higher extent than never smokers (data not shown).

Neither current nor past smokers were at an altered risk of breast cancer as compared to never smokers, regardless of smoking intensity, total smoking duration or total number of pack-years (Table 1). Neither smoking initiation in adolescence nor prior to first birth was significantly related to breast cancer risk, again regardless of whether the woman reported to be a current or past smoker (Table 1).

**Figure 1:**
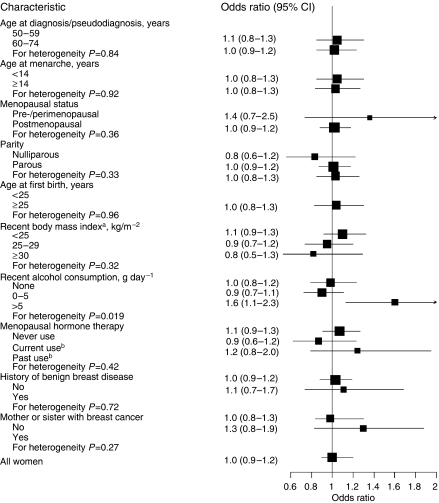
Relative risk of breast cancer for current smokers as compared to never smokers, by different characteristics. Black squares indicate odds ratios (ORs) area, which is proportional to the amount of information contributed (ie, to the inverse of the variance of the logarithm of the OR). Lines indicate 95% confidence intervals. All estimates were adjusted for age, age at first birth, recent body mass index and recent alcohol consumption when applicable. Recent denotes 1 year before data collection. (**A**) Women ever using menopausal hormone therapy excluded. (**B**) Current and past use defined as last use ⩽6 months and >6 months before diagnosis/pseudodiagnosis, respectively.

The relationships between breast cancer and current smoking did not vary significantly with levels of other characteristics, except for alcohol consumption (Figure 1). A similar pattern was seen with smoking initiated before first birth (data not shown). Lastly, current smoking as compared to never smoking among overweight postmenopausal women was not associated with any alteration in risk, even when smoking was commenced after the first birth (OR 1.0 (95% CI 0.6–1.7), based on 35 and 36 exposed cases and controls, respectively).

## DISCUSSION

We did not observe any association between smoking at any time period, duration or intensity, and the risk of breast cancer – either overall or among most subgroups. Yet, we found some evidence of an interaction between alcohol consumption and smoking.

Our null finding is consistent with results from a collaborative reanalysis including 53 studies (Collaborative Group on Hormonal Factors in Breast Cancer, 2002). This analysis was restricted to teetotallers, since the relationship between smoking and breast cancer was observed to be substantially confounded by alcohol consumption. We believe that residual confounding may explain our finding of a positive association between smoking and breast cancer among women who reported a high consumption.

Smoking has been proposed to evoke a dual effect on breast cancer risk (Terry and Rohan, 2002). Firstly, smoking is hypothesised to reduce breast cancer risk via an ‘anti-oestrogenic effect’ since smokers experience effects mimicking low endogenous oestrogen levels, like earlier menopause, lower relative weight, increased risk for oestoporos, decreased risk for endometrial cancer, as well as reduced levels of oestrone and oestradiol after menopausal hormone therapy. Yet, smoking does not seem to affect serum oestrogen levels in premenopausal or postmenopausal women (Key *et al*, 1991). Nevertheless, any ‘antioestrogen-mediated’ inverse association between smoking and breast cancer may be more pronounced among women with high endogenous or exogenous sex steroid exposure, including premenopausal women, postmenopausal women with overweight/obesity (Key *et al*, 2003) and users of menopausal hormone therapy. Yet, neither our data nor those from the pooled reanalysis support any protective effect of smoking among women with such characteristics (Collaborative Group on Hormonal Factors in Breast Cancer, 2002).

Smoking has further been proposed to initiate breast carcinogenesis through a genotoxic impact of tobacco-related compounds (Morabia, 2002). The breast epithelium is thought to proliferate rapidly during the period between menarche and first pregnancy and therefore to be especially vulnerable to malignant transformation (Russo and Russo, 1980). Corroborating these lines of reasoning, Band *et al* (2002) reported an increased risk with smoking initiated prior to first birth and among nulliparous women. However, a recent meta-analysis showed a null association between smoking prior to the birth of a first child and risk of breast cancer (Lawlor *et al*, 2004).

We tested the suggested hypothesis (Band *et al*, 2002) that smoking may reduce breast cancer risk among women through an ‘anti-oestrogenic effect’ – if not offset by a purported carcinogenic effect of smoking before first pregnancy. We could, however, not discern any impact of recent smoking among overweight/obese postmenopausal women who commenced smoking after their first birth.

A larger proportion of controls than cases were excluded from the analyses since 14% of all eligible controls participated via a telephone interview that did not elicit information on alcohol consumption. Sensitivity analyses evaluating the exclusion of such controls were, however, not indicative of any introduction of selection bias. Subject to debate, passive smoking has been proposed to increase breast cancer risk *per se* and to confound estimates of the relative risks for breast cancer associated with active smoking (Johnson, 2005). We did not collect information about passive smoking. Yet, the available evidence suggests that the effect (if any) of passive smoking may be confined to premenopausal women (Johnson, 2005; Miller *et al*, 2007). Since our study population is postmenopausal, we believe that confounding by passive smoking is an improbable explanation of our null findings. Lastly, such confounding appears unlikely since active smokers are indeed heavily exposed passive smokers.

In summary, results from this study do not support any association between active smoking and the risk of breast cancer.

## Figures and Tables

**Table 1:** Relative risk of breast cancer in relation to current and past smoking duration, intensity and initiation

**Smoking measure**	**Cases/controls**	**OR^a^ (95% CI)**	**Cases/controls**	**OR^a^ (95% CI)**
Never smoker	1526/1453	1.0 (ref)	1526/1453	1.0 (ref)
				
	**Current smokers**		**Past smokers**	
Ever smoker	657/589	1.0 (0.9–1.2)	601/541	1.0 (0.9–1.1)
				
*Duration of smoking, years*				
1–10	22/28	0.7 (0.4–1.3)	255/216	1.0 (0.8–1.3)
11–30	173/147	1.0 (0.8–1.3)	282/257	1.0 (0.8–1.2)
>30	457/406	1.1 (0.9–1.2)	61/65	0.9 (0.7–1.4)
*P* for linear trend^b^		0.58		0.95
				
*Cigarettes per day*				
1–10	282/259	1.0 (0.9–1.3)	373/349	1.0 (0.8–1.2)
11–20	347/297	1.1 (0.9–1.3)	196/170	1.0 (0.8–1.3)
>20	23/25	0.8 (0.4–1.4)	29/19	1.2 (0.7–2.2)
*P* for linear trend^b^		0.71		0.73
				
*Pack-years*				
1–10	114/114	0.9 (0.7–1.2)	391/359	1.0 (0.8–1.1)
11–20	193/187	1.0 (0.8–1.2)	118/103	1.0 (0.8–1.3)
21–30	229/153	1.4 (1.1–1.7)	61/59	0.9 (0.6–1.3)
>30	116/127	0.9 (0.7–1.2)	28/17	1.5 (0.8–2.8)
*P* for linear trend^b^		0.77		0.69
				
*Age at initiation, years*				
<20	359/281	1.2 (1.0–1.4)	300/265	1.0 (0.8–1.2)
20–29	196/204	0.9 (0.7–1.1)	221/202	1.0 (0.8–1.2)
⩾30	99/99	1.0 (0.7–1.3)	77/71	1.1 (0.8–1.5)
*P* for linear trend^b^		0.73		0.99
				
*Initiation in relation to birth*				
Prior	284/223	1.2 (0.9–1.4)	289/260	1.0 (0.8–1.2)
At around the same time	196/204	1.0 (0.8–1.2)	157/159	1.0 (0.8–1.3)
Following	89/99	1.0 (0.7–1.4)	72/63	1.3 (0.9–1.9)

a Adjusted for age (5-year categories), age at first birth (nulliparous, 20–24, 25–29, ⩾30 years), recent body mass index (in quintiles) and recent alcohol consumption (no intake, <5, 5<−10, >10 g day^−1^).

b *P* for Wald *χ*^2^, test of linear effect using the original continuous variable.
